# A Case Report of Enteric Fever Caused by Consumption of Lettuce

**DOI:** 10.7759/cureus.42948

**Published:** 2023-08-04

**Authors:** Noman Salih, Izhar Ullah, Hidayat Ullah, Numan Ghani, Muhammad Ihtisham

**Affiliations:** 1 Internal Medicine, Hayatabad Medical Complex Peshawar, Peshawar, PAK; 2 Internal Medicine, Lady Reading Hospital MTI Peshawar, Peshawar, PAK; 3 Internal Medicine, Khyber Medical Center, Peshawar, PAK

**Keywords:** and anti-bacterial agents, diarrhoea, appetite, dna topoisomerases, lettuce, ciprofloxacin, salmonella typhi, typhoid fever

## Abstract

When *Salmonella* *enterica* serotype Typhi (*S.* Typhi) is present in the water, food supply, or both, it leads to the rapid development of typhoid fever. Because lettuce is an ideal host for *S.* Typhi's survival, lettuce grown in animal manure can be the probable source of typhoid fever. Prompt identification and proper antibiotic treatment can lessen the burden of typhoid fever on the public health system. A male farmer, age 29, was admitted to our hospital with a serious major complaint of abdominal pain, lack of appetite, and runny diarrhea. The newly hospitalized patient had a 2-week history of high-grade fever, abdominal pain, loss of appetite, watery diarrhea, back pain, and generalized body aches. Blood culture is the most accurate test for the diagnosis of typhoid fever. Blood culture was positive and showed sensitivity to ciprofloxacin and other drugs. To cure the typhoid, 500 mg of ciprofloxacin was administered twice daily for 7 days. Pathogenic components, species that are infected, and host immunity all play a role in typhoid fever pathogenesis. Typhoid fever is common in underdeveloped countries due to tainted food or hazardous water sources. This report's main goals are to draw attention to the significance of food safety procedures and to the potential dangers of consuming raw vegetables.

## Introduction

Water and food contaminated with *Salmonella enterica* serotype Typhi (*S.* Typhi) are the main causes of typhoid/enteric fever [1]. Globally, typhoid/enteric fever is a leading cause of morbidity and mortality with the prevalence of culture-confirmed typhoid fever being 6.5% in the study conducted by Batire S et al. [2]. The primary clinical symptoms of typhoid/enteric fever are high-grade fever, chills, stomach discomfort, tachycardia, generalized body pain, back pain, and headaches [3]. Clinical samples such as blood, stool, urine, bone marrow, polymerase chain reaction, and the Widal test (obsolete now) are used in the most common laboratory diagnosis of typhoid/enteric fever [4]. Blood culture is the most specific test although time consuming. Ciprofloxacin was first prescribed to treat typhoid fever which works by preventing *S.* Typhi DNA topoisomerase and DNA gyrase from replicating DNA, which is how typhoid spreads [5]. Because lettuce is the preferred host for *S.* Typhi survival, this study adds new knowledge to the scientific awareness that lettuce cultivated in manures promotes typhoid disease. This case report details a rare instance of adult typhoid or enteric fever caused by lettuce consumption.

## Case presentation

A 29-year-old male farmer was admitted to the hospital with abdominal pain, appetite loss, and diarrhea as his main complaints for the last 2 weeks. The patient was hospitalized after presenting with heat, abdominal pain, loss of appetite, watery diarrhea, back pain, and generalized body aches over the previous 2 weeks. The patient has been farming in an area where manure has been used as fertilizer for the past 12 years. In the past, he had grown vegetables and crops in animal manure. After eating lunch at roughly 3 p.m., the patient started experiencing diarrhea at midnight. The symptoms did not improve after treatment with antidiarrheal and antipyretics for a few days. After worsening symptoms, he was transported to the hospital and was diagnosed with probable enteric fever based on his medical history, which revealed poor hygiene, fever, gastrointestinal pain, and lettuce consumption. His financial situation makes it clear that he must utilize animal manure to grow vegetables and other crops more quickly because he cannot purchase artificial fertilizers.

The patient grew lettuce this season since he loved it so much. The vegetables had recently become ripe enough to be sliced, eaten, or sold. He brought home some of the lettuce from work one day. He ate it for dinner, but after it had been left out in an open space within the house for a few days. Within 12 hours of consumption, the farmer exhibited the aforementioned clinical signs. We decided to identify the disease type through laboratory investigations and diagnostic criteria after the patient stated, "I had reason to believe typhoid fever because the lettuce I ate was grown on animal manure and not washed with fresh water after keeping it at home for some days."

Physical examination revealed a high-grade fever (105.4°F) (reference range: 98.6°F), a coated tongue, mild dehydration, heart palpitations, a heart rate of 112 beats per minute (reference range: 60-100 beats per minute), a breath rate of 14 breaths per minute (reference range: 12-16 breaths every minute), and a blood pressure reading of 118/78 mmHg (reference range: 120/80 mmHg). The results of the remaining tests were uneventful. Blood cultures were obtained and other laboratory investigations were performed. Table 1 lists the results of the laboratory investigations.

**Table 1:** Important laboratory investigations WBC, white blood cell; Hb, hemoglobin; PLT, platelets; Na, sodium; K, potassium; Cl, chloride; ALT, alanine aminotransferase; ALP, alkaline phosphatase

Test	Reference	Result
WBC (/µl)	4000 -11000	3200
Hb (gr/dl)	11.5 - 17.5	10.6
PLT (/µl)	150000 - 450000	311000
Amylase (IU/L)	<90	54
Bilirubin (mg/dl)	0.1 - 1	0.3
ALT (IU/L)	10 - 50	24
ALP (IU/L)	<390	118
Na (mmol/l)	135 - 150	137.9
K (mmol/l)	3.5 -5.1	3.4
Cl (mmol/l)	96 - 112	106

No malaria parasites were found in the thick and thin peripheral blood smear examination. An ultrasonographic examination of the abdomen and pelvis was done to rule out any local complication which was unremarkable. Laboratory investigations revealed lymphopenia and a decreased white blood cell count. The patient received paracetamol for fever and metronidazole for diarrhea and prevention of pseudomembranous colitis. The patient recovered within 7 days, and none of his relatives had the same symptoms.

On admission, ciprofloxacin 500 mg was started twice daily for 7 days to treat typhoid by suppressing the growth of *S.* Typhi DNA topoisomerase and DNA gyrase. Paracetamol 500 mg was administered three times daily for 7 days starting on the same day to reduce the temperature by preventing prostaglandin E2 secretion, which increases heat secretion and decreases heat loss. Most of the patient's symptoms disappeared after 6 to 7 days, except for a low-grade fever that was still present. Seven days after the order for blood culture, a blood culture report was also obtained. The culture report showed *S.* Typhi isolation after 7 days, which was sensitive to azithromycin, amikacin, imipenem, and ciprofloxacin. The culture report also reported resistance to cefoperazone/sulbactam, co-trimoxazole, cefixime, cefotaxime, and amoxicillin. The patient was hospitalized, treated for typhoid fever, and sent home with the necessary medication. On follow-up after 2 weeks, the patient was doing fine.

## Discussion

*S.* Typhi is the culprit behind typhoid/enteric fever [6,7]. Enteric fever is typically accompanied by fever, chills, tachycardia, stomach pain, watery diarrhea, loss of appetite, and weight loss [8]. Typhoid fever was also predicted using the aforementioned common clinical symptoms. Typhoid fever is caused by pathogenic components, infecting species, and host immunity [9]. The most frequently observed pathogenic characteristics of typhoid fever are dissemination to systemic areas, persistence and replication within host cells, and adhesion to and penetration of gut epithelial cells [10]. The significance of typhoid fever in the public health burden is diminished by prompt diagnosis and proper antibiotic therapy [11]. Typhoid fever infection decreased the number of packed cells by 27.4% (normal value: 38.0-47.5%) and leukocytes by releasing toxins in the bone marrow when the WBC count was 3200/mm3 (normal value: 4000-11000 cells/mm3) due to metabolic processes in Salmonella. The first to the third week following the commencement of the illness is when blood cultures are most important. Rapid therapy for the infection is aided by isolation, prompt identification, and accurate antibiotic sensitivity testing. The first-line treatments for enteric fever include chloramphenicol, ampicillin, and cotrimoxazole [12]. However, our patient was resistant to the first-line medications. Antibiotics that eradicate *S.* Typhi bacteria are the primary and only curative agents for typhoid fever [13]. Fluoroquinolones have become an accessible and preferred treatment for typhoid fever in many locations with limited resources [14]. By blocking the DNA topoisomerase and DNA gyrase of *S.* Typhi, ciprofloxacin (500 mg) was administered twice daily for 7 days to treat typhoid [15]. Since the mid-1980s, Multidrug-resistant typhoid fever has caused outbreaks in several countries in the developing world, resulting in increased morbidity and mortality, especially in affected children below five years of age and those who are malnourished [16]. By preventing the release of prostaglandin E2, which increases heat secretion and decreases heat loss, paracetamol 500 mg was administered three times per day for 7 days to reduce fever [17]. Hyperthermia, or increased body temperature, is also decreased by paracetamol [18].

## Conclusions

*S.* Typhi is the agent of typhoid/enteric fever, which is primarily a bloodstream infection. Typhoid diseases have been linked to travel histories in developing nations owing to tainted food and contaminated water supplies. The results of this study indicate that *S.* Typhi thrives well on lettuce grown in animal manure. Blood culture was used in the diagnostic process, which is the gold standard, although time-consuming, but helped in the isolation of *Salmonella* and the identification of sensitive drugs. Ciprofloxacin, a fluoroquinolone drug, is the most effective treatment for typhoid fever. Based on our case, physicians should suspect lettuce as a source of enteric fever, especially in endemic regions where manure is used as a fertilizer. After examining our case, physicians will be able to consider typhoid fever in farmers who consume lettuce and prevent its complications.
